# Anti-transgender rights legislation and internet searches pertaining to depression and suicide

**DOI:** 10.1371/journal.pone.0279420

**Published:** 2022-12-22

**Authors:** George B. Cunningham, Nicholas M. Watanabe, Erin Buzuvis

**Affiliations:** 1 Laboratory for Diversity in Sport, Department of Sport Management, University of Florida, Gainesville, Florida, United States of America; 2 Department of Sport and Entertainment Management, University of South Carolina, Columbia, South Carolina, United States of America; 3 School of Law at Western New England University, Springfield, Massachusetts, United States of America; University of Botswana, BOTSWANA

## Abstract

The purpose of this study was to examine whether anti-transgender rights legislation among state legislators is associated with increased suicide- and depression-related Internet searches. Employing a quasi-experimental non-equivalent control group design, we focused on bills that were introduced to state legislatures from July 2019 to July 2020. As our panel is constructed of 51 states/territories over a 52-week time frame, our final dataset is composed of 2,652 observations. Results showed that states’ passing of anti-transgender rights bills were linked with suicide- and depression-related Internet searches. Second, introducing or debating the bills did not have an association with Internet searches. Third, the defeat of anti-transgender bills was linked with fewer depression-related searches. Finally, the LGBT context in the state affected the results: anti-transgender legislation had a particularly strong association with suicide-related Internet searches when the state had a high LGBT population density.

## Introduction

In 2017, Texas was one of many states to consider some form of a bathroom bill-type legislation that would restrict the rights of transgender to use public restrooms congruent with their gender identity and expression [1]. Though there was strong support in the Texas Senate, the bill never reached the floor of the House of Representatives, largely because then-Speaker Joe Straus (a Republican) worried about the psychological impact of the bill on Texas residents. He commented, “I’m not a lawyer, but I am a Texan. I’m disgusted by all this…. *I don’t want the suicide of a single Texan on my hands*” (emphasis added, as cited in [2]).

Straus’s comments bring to the fore the potential link between legislation curtailing transgender rights and decreased psychological health. These are real concerns given that transgender individuals, especially transgender youth, report more attempted suicides than do their peers [3, 4]. Further, there is some evidence that organizational, community, and state-level policies can all influence the psychological health of stigmatized groups, though most of the scholarship to date has focused on lesbian, gay, and bisexual individuals. For instance, a survey of over 31,000 teens in Oregon (USA) showed that anti-bullying policies by the schools were linked with fewer attempted suicides by lesbian and gay youth [5]. The effects are not just limited to youth, though. Lesbian, gay, and bisexual adults living in states that do not prohibit discrimination based on sexual orientation report more psychological disorders than do their peers in more inclusive states [6]. Further, the introduction of restrictive laws is associated with worsening psychological health for LGB individuals [7].

Though comparatively scarcer than scholarship related to lesbian, gay, and bisexual individuals, there is emerging evidence pointing to the relationship between restrictive laws and policies, and transgender people’s health. Anecdotally, reports from a suicide hotline indicate an almost doubling of calls from transgender youth during the time that the 2017 Texas bill was debated [8]. When President Trump introduced a prohibition on transgender individuals serving in the military [9], suicide hotline calls increased further (see also [10]). Moving to the limited empirical scholarship, Perez-Brumer, Hatzenbuehler, Oldenburg, and Bockting found that structural forms of discrimination based on *sexual orientation* were related to increased lifetime suicide attempts by *transgender* individuals [11]. More recently, DuBois, Yoder, Guy, Manser, and Ramos analyzed state-level laws affecting transgender individuals and their association with multiple health measures. They found that transgender restrictions were linked with poorer mental health, less frequent doctor visits, and greater alcohol use among transgender residents [12]. These findings support Winter and colleagues’ conclusion that “laws and legal reform play an important part in ensuring social inclusion, with consequent effects on the health and wellbeing of transgender people” (p. 320) [13].

Our review suggests that (a) state-level laws and policies can influence the health and well-being of its residents, especially those from underrepresented and marginalized groups; and (b) analysis of how state laws can influence the health of transgender individuals is a still nascent field of scholarship. The purpose of this study was to examine whether anti-transgender rights legislation among state legislatures is associated with increased suicide- and depression-related Internet searches. In conducting this study, we contribute to small but growing body of research examining the role of structural determinants on health outcomes. Our study design also moves beyond the cross-sectional nature of previous research on the topic: as explained in more detail in the Methods, we compared Internet searches prior to and after the bill was debated and made comparisons in states where such legislation was not considered. Thus, we have more confidence in the directionality of the findings. In the following space, we overview the theory guiding our work and then provide a study overview.

## Theoretical framework

### Stigma

We ground our work in stigma theory. People who are stigmatized have characteristics (or others believe that possess) characteristics that mark them as different than others and therefore devalued [14]. Stigmas can take several forms and vary in their visibility to others, controllability, the degree to which they affect one’s appearance, behaviors, or group membership [14]. They also vary by time and culture, such that what is considered stigmatizing in one context can (and frequently does) vary in another. As a result, the legal protections for those who are stigmatized also varies by time and place [15]. Ultimately, through prejudices, stereotyping, and discrimination, stigma serves as a form of social control [16].

Stigma against transgender individuals can manifest at individual, interpersonal, and structural levels [17, 18]. Stigma at the individual level includes feelings people have about themselves, as well as the beliefs they think others have about them. At the interpersonal level, stigma takes on direct and enacted forms, such as harassment, discrimination, violence, rejection, and assault of transgender individuals. Finally, structural forms of stigma reflect the norms, customs, and policies that negatively affect transgender people. Examples include stigmatizing laws and ordinances, a lack of healthcare access, economic inequalities, and gender inequalities, among others. Hughto et al. wrote, “structural, interpersonal, and individual forms of stigma are highly prevalent among transgender people and have been linked to adverse health outcomes including anxiety, suicidality, substance abuse, and HIV” (p. 223) [18].

As our study is concerned with the effects of anti-transgender rights laws, we specifically focus on structural forms of stigma and their impact. Power is a key element of structural stigma, as people in the stigmatizing majority use it to exclude and subjugate transgender individuals [18]. The laws, polices, and ordinances send a consistent message that the stigmatized group is different, the other, and less than. Over time, these repeated messages can result in a form of minority stress, whereby distal or external stressors negatively impact the lives of transgender people [19]. These stressors ultimately manifest in health disparities, whereby transgender individuals are at higher risk for adverse physical and psychological health, relative to their cisgender counterparts [20].

### Suicide searches

As the preceding discussion illustrates, structural stigma negatively affects transgender individuals’ mental health and wellbeing. Previous anecdotal evidence also suggests a link between heightened structural stigma–that is, legislators debating bills that would curtail transgender rights–and individuals seeking suicide information and help [8, 10]. Beyond calling helplines, people might also seek suicide-related information online.

Some might search for suicide-related terms as a precursor to them taking their own lives. Stephens-Davidowitz argued that people conduct Internet searches in what they believe to be relative private and anonymous, and thus, they are likely to seek information that they might not otherwise share or admit to [21]. If this is the case, then online searches for suicide information might be predictive of actual suicide behavior. In one of the early studies exploring this topic, McCarthy examined Google search activity from 2014–2019 and found that searches for suicide-related terms were linked with Centers for Disease Control statistics for suicides and self-harm [22]. The relationship was significant and positive for younger individuals, but not the general population. Others have observed similar associations in various contexts [23, 24].

Whereas Internet search data provide information about what people from a specific area search for, there are also potential limitations. For example, the researcher does not know who is conducting the search or their motivation. People might search for suicide-related terms as a precursor to related behaviors, but they might also search for other reasons, too, including resources and support. They might seek support groups, hotline information, or other preventative measures. The lack of intent associated with Internet searches might explain why some authors have observed a null effect between suicide Google searches and subsequent behaviors [25]. Still others have found that other terms, such as those related to anxiety, sleep, and unemployment are more predictive of suicide behavior than searches of suicides, specifically [26].

This evidence suggests that, irrespective of the motive–that is, whether the search is to facilitate self-harm or to seek preventative resources, an Internet search for suicide-related information is associated with distress and worry. Thus, increased searches in the time surrounding anti-transgender legislation, would suggest that the legislation serves as a form of structural stigma that spurs worry, anxiety, and the potential for self-harm among transgender individuals.

### Context of current study

As previously noted, the purpose of this study was to examine whether debating anti-transgender rights legislation among state legislators is associated with increased suicide-related Internet searches. We focused on bills that were introduced to state legislatures from July 2019 to July 2020. Specifically, we considered all bills that were listed by the Anti-Transgender legislative tracker [27], as well as the status of the bill in terms of when they were introduced, whether they reached committee, if they passed or failed in the legislative process, as well as when they were signed into law by the governor if they were passed. To check these data, we then cross-checked all of the bills with those posted by the American Civil Liberties Union (ACLU) on their anti-transgender bill tracker. Any bill that was not included in both datasets was then checked by looking at the actual legislation posted on each state’s respective legislation site. Those bills that did not contain anti-transgender language were then removed from the data set. Overall, during this time period, a total of 40 bills were introduced and reached committee, while three of these bills were passed and signed into law. Examining the data in Table 1, it is evident that anti-transgender bills were introduced in 17 states during the time period we analyzed, with one state (Idaho) having passed a vote and signed such legislation into law.

**Table 1:** Anti-transgender bills by state.

State	Number of Bills Introduced	Number of Bills in Committee	Number of Bills Passed	Number of Bills Signed
Alabama	3	3	0	0
Arizona	1	1	0	0
California	1	1	0	0
Colorado	2	2	0	0
Florida	2	2	0	0
Idaho	2	2	2	2
Kansas	1	1	0	0
Kentucky	1	1	0	0
Louisiana	1	1	0	0
Mississippi	4	4	0	0
Missouri	8	8	0	0
New Hampshire	2	2	0	0
New York	0	0	0	0
Ohio	2	0	0	0
South Dakota	1	0	0	0
Tennessee	8	7	0	0
Utah	1	1	0	0
**Total**	**40**	**36**	**2**	**2**

By combining this anti-transgender legislative data with the Google Trends information by state for keywords related to mental health it allows us to examine whether the introduction or passage of anti-transgender bills has a relationship with individuals searching for terms such as suicide and depression. Considering the previously discussed theorization on the stigmatization of identifying characteristics and the potential impacts it may have on mental health, we formulate two research questions.

**Research Question 1**: Is there a relationship between the introduction of anti-transgender bills and Google searches for “suicide” or “depression”?
**Research Question 2**: Is there a relationship between the passage of anti-transgender bills and Google searchers for “suicide” or “depression”?

As previously noted, researchers focusing on Internet searches do not know who is conducting the search or their characteristics. In the context of the current study, we do not know the gender identity of the person online. We can partially account for these shortcomings by considering the characteristics of the community, overall. Thus, in pursuing the research questions, we also examine the influence of the share of people in the state who identify as LGBT. If anti-transgender legislation negatively relates to transgender people’s mental health, then the searches should increase in areas with a high share of LGBT people.

Examining these research questions will not only provide important information of the effect that the creation of anti-transgender laws has on the mental health of individuals, but also critical policy implications regarding protecting those individuals who could be adversely impacted by such legislation.

## Method

To examine the research questions focused on the relationship between anti-transgender bills and Google searches for certain keywords related to mental health, we utilized a quasi-experimental non-equivalent control group design. Specifically, a panel data set was created by collecting data for all 50 states and the District of Columbia over a 52-week (one-year) time period ranging from the end of July 2019 to the beginning of July 2020. This time frame was chosen as it encompassed a varying number of bills in states from various regions across the U.S., allowing for the comparison of whether those states that were introducing, passing, and signing anti-transgender bills into law had an effect on Google searches. The use of this one-year sample also provided the ability to include time trends within the data set, thus allowing the consideration of the impact of legislation over time. As our panel is constructed of 51 states/territories over a 52-week time frame, our final dataset is composed of 2,652 observations (51 × 52 = 2,652). The data are available at https://figshare.com/articles/dataset/LGBTQ_Legislation_Data_Plos_One/20352921.

To begin, two dependent variables were created for this study. The first dependent variable is a measure of the number of Google searches for the word “suicide” for each state/territory in each week, while the second dependent variable substitutes the term “depression” for “suicide.” To gather these data, we utilized the Google Trends data site, that provides information in terms of the volume of searchers within a given geographic area on a weekly basis [28]. Prior scholars have noted that while Google Trends provides a rich source of information to analyze what individuals in each region are searching for, the data from the site cannot simply be downloaded and compared across regions [29]. Rather, there is a need to normalize the data across regions following the approach developed by Stephens-Davidowitz [21], which can be accomplished using common search words that are universal across all areas, such as “weather.” To develop our dependent variable, we first collected the weekly volume of searches for “suicide” and “depression” for each state. Following this, we then normalized this measure by using the values produced by two additional searches, one for the common search term “weather” and another using the combination of both the keyword and common search term (“suicide + weather”), as has been done by prior scholars [21, 26, 28]. Following this, we then normalized the measure of Google searchers for suicides for each week in every market, by using the following equation:

Suicide=Suicide−(Weather−(Suicide+Weather)) (1)

This same process was then repeated by replacing the term “suicide” with “depression.” Finally, because of the non-normal distribution of the normalized search term across the dataset, we then transformed both dependent variables by their natural logarithm, as is commonly done in econometric studies examining Google Trends data [30]. Additionally, transforming the dependent variables in this manner enhances the understanding of the results, as the coefficients produced from estimation can be interpreted in the percent change.

Turning to the independent variables, we first develop a series of measures to control for the various stages and volume of anti-transgender legislation that was being passed through each state in every week. To begin with, we develop a continuous variable *Bill* that measures the number of anti-transgender bills being considered by a state in a week. In this, we define that consideration of a legislation as a bill being formally proposed, introduced, in committee, being voted on, or passed within the observed week. Next to consider the overall volume of anti-transgender bills being introduced, we developed the measure *Introduced*, which is simply the count of the number of bills that were in their introductory phase to the state legislature in that week. Moving to the next phase of the legislative process, the *Committee* variable measures the number of anti-transgender bills that were in committee in a given state in each week. Next, we also created a measure of the number of bills that were passed in a given week in a state (*Passed*) and the number that failed (*Failed*).

Because legislation can often take many weeks for a bill to be introduced, considered, and then voted on by a legislature, we develop a time trend variable to measure how long bills have been introduced and considered by each state government. Specifically, we create a trend variable *BillTrend* the number of weeks that anti-transgender bills have been under consideration. Additionally, we also include the square term of this trend variable (*BillTrendSq*) so as to consider whether any potentially impact of the trend may be increasing or decreasing over time. From this the formal model for out study takes the form of:

$${Suicide}_{it}=\alpha_{0}+\beta_{1}{BillStatus}_{it}+\beta_{2}{BillTrend}_{it}+\beta_{3}{BillTrendSq}_{it}+{\beta_{4}{Introduced}_{it}+\beta_{5}{Committee}_{it}+\beta }_{6}{Passed}_{it}+\beta_{7}{Failed}_{it}+\mu_{it}$$ (2)

where *i* indicates states and *t* indicates weeks. The summary statistics for the variables included in the model can be found in Table 2.

**Table 2:** Summary statistics.

Variables	Avg.	Std. Dev.	Min	Max
Bill	0.2783	1.111	0	8
BillTrend	1.545	5.717	0	52
BillTrendSq	35.05	191.69	0	2704
Introduced	0.0098	0.0985	0	6
Committee	0.1497	0.7576	0	8
Passed	0.0008	0.0388	0	2
Failed	0.0121	0.2454	0	8

In order to estimate the results from the model presented in Eq 2, we utilize multiple regression analysis. Due to the panel nature of our dataset, there is need to consider various potential econometric issues that may arise in the estimation of our results. One of the main issues in estimating results from panel data when using regression analysis is whether to use fixed or random effects [31]. To consider which type of effect is suitable for the final estimation of the results, we estimated a fixed-effects and random-effects regression from our base model, and then calculated a Hausman test using these generated estimates. The results from the Hausman test were significant (*p* = 0.003), suggesting that a fixed-effects regression is the appropriate methods of estimation that should be utilized to produce our results [31]. Another potential issue that could bias the results would be the presence of heteroscedasticity within our dataset. In response to this, we conducted the Breusch-Pagan/Cook-Weisberg tests, which was significant at the five-percent level for all of the models we utilized in this study. To correct for this, we utilized the common econometric approach of clustering standard errors by state, which accounts for the possibility of correlation amongst standard errors [32]. Thus, the final results for this study were estimated using a fixed-effects panel regression with standard errors clustered by state. Additionally, it is important to note that the fixed effects models used to estimate the results within this study drop all time invariant variables in their reported results. That is, a fixed-effect is included for each state that captures a range of variables whose values do not change over the duration of the dataset, which includes factors such as unemployment rate, population, and so forth. Thus, while factors have been shown in previous research to have an impact on suicide rates and depression, they are not included within this study because of the nature of the models and the dataset.

## Results

To answer our two proposed research questions, we estimated a total of eight regression models. The first four models (Table 3) were estimated using the natural log of Google searches for the word “suicide” as the dependent variable, while the last four models (Table 4) utilized the natural log of Google search for the word “depression.” For each dependent variable, four variations of our base model were estimated considering different ways through which to measure the progression of time. That is, the first and second model utilize the variable *Bill* to simply measure the number of bills being considered at any given time, while the third and fourth model use *BillTrend* and *BillTrendSq* in order to consider whether the length of time that anti-transgender bills were with the legislature had an impact on the number of searches for “suicide” or “depression.” Additionally, as the volume of searches for these keywords could potentially change over the course of the year, we recognized that there might be the need to consider different periods of time in the fixed-effects within our panel data. As such, we estimated each group of models with either a week fixed-effect or a month fixed-effect. Tables 3 and 4 display the variables used within each model, as well as the type of time fixed-effect that was used when estimating each model.

**Table 3:** Regression results (dependent variable = natural log of searches for “suicide”).

Variables	Model 1	Model 2	Model 3	Model 4
Bill	0.0187	0.0119	---	---
	(0.0192)	(0.0191)	---	---
BillTrend	---	---	0.0011	0.0001
	---	---	(0.0021)	(0.0020)
BillTrendSq	---	---	<0.0001	<0.0001
	---	---	(<0.0001)	(<0.0001)
Introduced	-0.0067	0.0033	-0.0020	0.0065
	(0.0141)	(0.0139)	(0.0134)	(0.0142)
Committee	-0.0038	-0.0033	-0.0041	-0.0028
	(0.0053)	(0.0053)	(0.0042)	(0.0042)
Passed	0.1678***	0.1293***	0.1726***	0.1329***
	(0.0258)	(0.0101)	(0.0248)	(0.0085)
Failed	0.0097	0.0022	0.0097	0.0026
	(0.0063)	(0.0065)	(0.0074)	(0.0074)
Week FE	Yes	No	Yes	No
Month FE	No	Yes	No	Yes
R-Squared	0.3701	0.2190	0.3701	0.2191

**Table 4:** Regression results (dependent variable = natural log of searches for “depression”).

Variables	Model 5	Model 6	Model 7	Model 8
Bill	-0.0202**	-0.0226**	---	---
	(0.0244)	(0.0244)	---	---
BillTrend	---	---	-0.0017***	-0.0017***
	---	---	(0.0025)	(0.0025)
BillTrendSq	---	---	0.0001***	0.0001***
	---	---	(<0.0001)	(<0.0001)
Introduced	0.0015	0.0040	-0.0011	-0.0011
	(0.0111)	(0.0117)	(0.0084)	(0.0084)
Committee	0.0041	0.0074	0.0031	0.0031
	(0.0054)	(0.0061)	(0.0063)	(0.0063)
Passed	0.0500***	0.0458***	0.0451**	0.0451***
	(0.0221)	(0.0273)	(0.0292)	(0.0292)
Failed	-0.0100	-0.0173**	-0.0188	-0.0188**
	(0.0072)	(0.0067)	(0.0065)	(0.0065)
Week FE	Yes	No	Yes	No
Month FE	No	Yes	No	Yes
R-Squared	0.3953	0.2675	0.3961	0.2679

Turning to the results from Table 3 focused on the volume of Google searchers for the term “suicide,” all four models returned similar results. Specifically, *Passed* was positive and significant at the one percent level, indicating that when bills were passed it lead to an increase in the volume of searches on Google for the word “suicide.” As *Passed* is a continuous variable measuring the number of anti-transgender bills passed within a state, the coefficient indicates the percentage increase in searches that occurred in that week. Notably, the coefficients ranged from 0.1293 to 0.1726, indicating that the passage of a single bill led to around a 13 to 17 percent increase in the volume of searchers for the word “suicide” within that state. In the case where a state passed two bills within the same week, the findings suggest that searches for “suicide” on Google could potentially increase by 26 to 34 percent in that time period.

Further considering the results from the first four models (Table 3), it is evident that none of the other variables had a significant relationship with the volume of Google searches for “suicide.” That is, the number of bills being introduced, in committee, and that failed did not have any impact on individuals searching for the term “suicide.” These results held consistent across all four models, even when using different time based fixed-effects or trend variables.

Turning to the second set of models (Table 4) that focused the volume of Google searches for the term “depression,” the number of bills passed in a state had a positive and significant relationship with searches for depression in all four models. Additionally, the number of failed bills had a negative and significant relationship with the volume of Google searches for “depression” in that week in the models using month fixed-effects (Models 6 & 8). Specifically, the coefficients highlight that for every anti-transgender bill passed in a week, there was about a five percent increase in searches for the word “depression.” At the same time, for a bill that failed, there was about a 1.0 to 1.9 percent decrease in the number of searches for depression. These findings differ slightly from the models from Table 3 focused on suicides, as while the number of bills passed impacted searches for both suicides and depression, the number of failed legislations only impacted Google searches for depression. Finally, while most of the other variables were insignificant, the number of bills and the bill time trends had significant relationships with searches for depression. Specifically, the results from Models 7 and 8 suggest a curvilinear relationship, where the longer a bill was under consideration by a state legislature the greater the number of searches there were for the term depression on Google. Finally, the status of bills as being introduced or in committee did not have an impact on individuals searching for keywords related to mental health on Google, this finding could potentially be attributed to the fact that the time trend is essentially capturing the movement of a bill through the legislature.

In addition to the models displayed in this study, additional models were estimated using different forms of the variables measuring the status of bills, including dummy variables to measure bill status, and additional measures denoting specific characteristics of each legislation. In these models, the dummy variable to measure bills simply captured if there was any anti-transgender bills being moved through the legislative process, and did not account for the actual volume of bills that were under consideration. Overall, the results held consistent across these models, with only the passage or failure of legislation being linked to changes in the volume of Google searches for “suicide” or “depression.” As such, the models displayed within this research, as well as our additional tests highlight the robustness of these findings.

Returning to the first research question focused on whether the introduction of anti-transgender bills had an impact on Google searches for “suicide” or “depression” within a region, the findings from all of the models indicate that no such relationship exists. That is, simply having a bill be introduced does not seem to have any effect on searches for keywords related to mental health. In regards to the second research question, the results from all eight models showed that the passage of anti-transgender bills increased the number of Google searches for both “suicide” and “depression.” As such, the findings from our estimated models highlight that the passage of such legislation can impact the volume of internet searches within a region in relation to select mental health related keywords. Finally, it should be noted that while there was an increase in Google searches for “suicide” or “depression” when anti-transgender bills were passed, there was likewise a decrease in “depression” searches when bills failed.

### Robustness check

In order to further consider the results of the models presented in Tables 3 and 4, additional regressions were estimated as a robustness check. Specifically, as the fixed-effects models prevent the inclusion of time-invariant variables, it prevents us from analyzing the impact that the size of the LGBT population of each state may have on determining the number of Google Trend searches for suicides/depression. That is, because the legislation directly impacts individuals from the LGBT community, it would likely be the case that states that have a higher number of people who identify as LGBT in a state would potentially lead to an increase in Google Trend searches for mental health keywords. Where in the previous models (Tables 3 and 4) the size of the LGBT population would be captured by the fixed-effect for each state, we utilize a different approach to allow for the inclusion of a new variable (*Population*) which measures the LGBT population of each state.

Because a fixed-effects panel regression will omit the results of any model that includes the *Population* variable, we instead utilize a random-effects regression. Although the results of our Hausman test suggested that a fixed-effects model was more suitable for our model and data, subsequent estimations of the models in Tables 3 and 4 using either fixed- or random-effects returned relatively similar results in terms of significance levels and coefficients. Thus, to further probe the results of our data, we introduce the *Population* variable into the model. Moreover, to consider the potentially moderating effect between the size of the LGBT population and anti-LGBT legislation, we also include an interaction between *Population* and the variable measuring the number of bills that made it to committee in the state legislature (*Committees*). Specifically, this interaction variable (*Com x Pop*) takes the form of the two variables multiplied by other another, and is included within the results for the random-effects regressions which are displayed in Table 5. For this robustness check, we focus on the interaction of *Population* with the number of committees, as the other variables measuring the progress of anti-transgender bills through the legislative process do not have enough variation to be able to properly estimate meaningful results from the interaction.

**Table 5:** Robustness checks using random-effects regressions.

	DV—Suicide	DV—Depression
Variables	Model 10	Model 11
Bill	0.0155	-0.0396
	(0.0195)	(0.0205)
NumIntroduced	-0.0087	0.0104
	(0.0151)	(0.0107)
Committees	-0.0217**	0.0026
	(0.0108)	(0.0122)
Population	<0.0001***	<0.0001***
	(<0.0001)	(<0.0001)
Com x Pop	<0.0001***	<0.0001
	(<0.0001)	(<0.0001)
Passed	0.2030***	0.0380***
	(0.0160)	(0.0137)
Failures	0.0097	-0.0073
	(0.0061)	(0.0077)
R-Squared	0.3138	0.3770

Examining the results, the two models (Model 9 and 10) both returned relatively similar results for Google Trend searches for suicide or depression. Notably, the existence of a bill, and the number introduced were insignificant in all models, which was similar to the findings from the previous models examining the number of searches for the term suicide. Interestingly, the number of bills in committees had a negative and significant relationship with the number of suicide searches, while it did not impact the number of searches for depression on Google. Next, the measure of LGBT population in a state was positive and significant in both models, indicating that those areas that had higher concentrations of people identifying as LGBT were more likely to have a greater volume of searches for keywords related to mental health. Continuing to the interaction effect of the number of bills in committee and the LGBT population of a state (*Com x Pop*), the interaction variable was positive and significant in relation to searches for suicide, and had no statistical relationship with depression searches. Overall, these findings suggest that the progress of a bill through the legislative process could have a profound impact, especially in areas where there is a higher population of people who identify as LGBT. Finally, like previous models, having bills being passed into law was positive and significant in relation to searches for mental health keywords, while the failure of a bill did not have an impact on searches.

## Discussion

The purpose of this study was to examine whether anti-transgender rights legislation among state legislatures is associated with increased suicide-related Internet searches. Our quasi-experimental non-equivalent control group design yielded several interesting findings. First, states’ passing of anti-transgender rights bills were linked with suicide- and depression-related Internet searches. Second, introducing or debating the bills did not have an association with Internet searches. Third, the defeat of anti-transgender bills was linked with fewer depression-related searches. Finally, the LGBT context in the state affected the results: anti-transgender legislation had a particularly strong association with suicide-related Internet searches when the state had a high LGBT population density. In the following space, we discuss contributions, implications, limitations, and future directions.

### Contributions

Our study makes several contributions to research and practice. First, we grounded the study in stigma theory, arguing that laws curtailing people’s rights send a signal that their identities are deviant, inappropriate, or “the other” in some way [14–16]. As a result, people who are targets of the legislation might experience stress and decreased wellbeing. Indeed, previous researchers have shown that restrictive laws and policies are related to destructive health behaviors on the part of transgender individuals [12]. We extended this research by showing that the passage of anti-transgender bills is linked with Internet searches related to depression and suicide. We *do not know* who was conducting the searches, nor do we know if people conduct the searchers to identify ways to complete suicide or to prevent that behavior. We *do know*, however, that (a) as the LGBT population in a state increased, so too did relevant searches, and (b) whatever the motivation, the increase in searches signals distress among people in the states where laws are passed–a pattern that has otherwise been unexplored (see also [33]).

Our research design is another contribution to the extant literature. Whereas other researchers have largely relied on cross-sectional designs, we devised a quasi-experimental non-equivalent control group study. Because randomization of state legislation would not be possible–making a true experiment equally impossible–our quasi-experimental design allowed us to conduct one of the most rigorous tests to date.

### Implications

Our findings have implications for political and legal advocacy for transgender rights, as well as providers of mental health care and other support to transgender communities. The increase in suicide-related Internet searches that correspond to the passage of anti-transgender laws suggests that the legislation induces stress responses, especially among people in states with high LGBT populations. Political advocates who oppose discrimination against transgender individuals can use this information in support of arguments that laws restricting their rights have another layer of costs in the form of stress and mental health impairment on the part of the community being targeted. In many states that have recently considered anti-trans laws, legislators have been unable to identify the harm they wish to prevent by restricting the rights of transgender people [34]. Especially when the perceived value of a proposed law is low (by virtue of being abstract and hypothetical), counterarguments that emphasize the present-day, concrete, and compelling harm to target populations that result from the passage of such laws produce should be persuasive in the legislative chamber and in the court of public opinion. As we noted in the Introduction, legislators already make this argument. The findings presented here make that argument more persuasive.

Legal advocates may also find these findings relevant in the fight to challenge anti-trans laws in court. Stigma has been a relevant consideration in cases challenging discrimination as a violation of the Constitution’s Equal Protection Clause. In *Brown v*. *Board of Education*, the Supreme Court famously relied on social science evidence that suggesting segregated schools stigmatized black children and produced negative psychological results, and courts have considered stigma in other cases as well. While stigma is not itself a conclusive factor of what makes a law unconstitutional, legal advocates can use the stigmatizing effect of laws that single out groups for differential treatment to help make that case. Our findings, which deepen our understanding of stigma deriving from laws that target transgender individuals, could thus be useful to those seeking to challenge anti-trans laws in court.

Finally, health care providers, social workers, teachers, and other professionals who provide support for transgender individuals should be aware that the passage of anti-trans legislation elevates internet searches about suicide. Those who serve in these roles likely already understand the minority stress model and the negative mental health outcomes associated with the experience of discrimination itself and work to provide appropriate clinical and other supportive response [19, 35]. Our findings suggest that mental health of people in a state is threatened not just by the occurrence of discriminatory acts, but to the passage of stigmatizing laws as well, and suggests that the mental health interventions used already should adapted to response to anti-trans legislation. Given that most of the anti-trans bills on the docket today are addressing the rights of students, teachers, and support providers in the school community should be particularly aware of this need.

### Limitations and future directions

Though our study makes several contributions, we also note potential limitations. First, though our quasi-experimental design is a strength relative to other studies on the topic, we recognize that it is not a true experiment. Thus, we cannot make claims of causation. Second, we have shown that passage of restrictive laws is linked with more depression- and suicide-related searches. We do not know, however, the nature of the searches. Thus, we can only note that the laws’ passages are associated with elevated searches concerning psychological wellbeing. Further, we examined one calendar year. As a result, we do not know if these patterns have persisted over time. One additional limitation of this work is the fact that during the course of the dataset, there are many weeks where there is little or no variance in the variable measuring whether legislation had been passed, as this was a relatively rare event. As such, because of the lack of variance for this measure, it is difficult to know the actually impact that the passage of bills had on searchers for the mental health keywords examined within this study. Finally, we collected data during the COVID pandemic, which also negatively affected people’s health. We tried to account for these possibilities through our fixed effects model.

Finally, we identify several future directions. As anti-transgender legislation is debated and passed, more work is needed to understand the effects on those targeted. In addition to the immediate health outcomes, for example, how does legislation targeting students and youth impact their persistence in school and long-term wellbeing. Second, what are factors predictive of anti-transgender laws passing (as opposed to being debated but ultimately failing). Finally, researchers have used Google search data to explore many outcomes, including suicide, racial animus, and COVID-19 spread, among others [21, 22, 25, 30, 33]. We encourage further exploration of this data source to explore other novel associations.
